# Unconditional Authentication Based on Physical Layer Offered Chain Key in Wireless Communication

**DOI:** 10.3390/e24040488

**Published:** 2022-03-30

**Authors:** Shaoyu Wang, Kaizhi Huang, Xiaoming Xu, Xiaoyan Hu, Jing Yang, Liang Jin

**Affiliations:** Wireless Communication Technology Office, Information Engineering University, Zhengzhou 450002, China; shaoyuwang_ndsc@163.com (S.W.); huangkaizhi@tsinghua.org.cn (K.H.); ee_xiaomingxu@sina.com (X.X.); ndscpls@163.com (X.H.); wanzheng18@alumni.hust.edu.cn (J.Y.)

**Keywords:** unconditional authentication, physical layer key generation, wireless communication

## Abstract

Authentication is a critical issue in wireless communication due to the impersonation and substitution attacks from the vulnerable air interface launched by the malicious node. There are currently two kinds of authentication research in wireless communication. One is based on cryptography and relies on computational complexity, the other is based on physical layer fingerprint and can not protect data integrity well. Both of these approaches will become insecure when facing attackers with infinite computing power. In this paper, we develop a wireless unconditional authentication framework based on one-time keys generated from wireless channel. The proposed unconditional authentication framework provides a new perspective to resist infinite computing power attackers. We study the performance of the unconditional authentication framework in this paper. First, a physical layer offered chain key (PHYLOCK) structure is proposed, which can provide one-time keys for unconditional authentication. The physical layer offered chain keys are generated by XORing the physical layer updated keys extracted from the current channel state information (CSI) and the previous chain keys. The security of PHYLOCK is analyzed from the perspective of information theory. Then, the boundary of the deception probability is conducted. It is shown that unconditional authentication can achieve a probability of deception $2^{-\frac{1}{2}H\left(k\right)}$, where $H\left(k\right)$ is the entropy of the one-time key used for one message. Finally, the conditions for unconditional authentication are listed. Our analysis shows that the length of the key and the authentication code need to be twice the length of the message and the encoding rules of the authentication code need to satisfy the restrictions we listed.

## 1. Introduction

### 1.1. Physical Layer Authentication

Authentication is one of the two important aspects of information security, and the other is known to be confidentiality [1,2]. Especially in wireless communication, due to the broadcasting characteristics of wireless communication and the improvement of the malicious adversary’s ability, wireless nodes are very vulnerable to impersonation and substitution attacks [3]. In an impersonation attack, the malicious adversary impersonates the transmitter and sends fraudulent information to the receiver when in reality nothing has been sent by the transmitter. While in a substitution attack, the malicious adversary intercepts legitimate message from the transmitter and successfully replaces the legitimate message with a fraudulent one [4,5]. With the rapid development of wireless communication and the increasing demand for communication security, authentication in wireless communication is becoming more and more indispensable [6]. Most of the existing wireless authentication approaches are based on computational complexity and become insecure when facing attackers with infinite computing power. In this paper, we propose an unconditional authentication framework to resist these attacks. Next, we will introduce the concepts and principles of authentication and the contributions of our paper.

Authentication needs to achieve two goals [7,8]. One goal is to ensure the integrity of the message. If the message received by the receiver is consistent with the message transmitted by the transmitter, the integrity of the message is achieved. However, merely achieving the integrity of the message is not sufficient, because the identity of the transmitter may be fake. For example, the receiver can confirm that the message is integral by the corresponding digest value, but cannot confirm who sent the message. Therefore, another goal is to confirm the identity of the transmitter. The receiver confirms that the message is from a legitimate transmitter by sharing the same secret key, which is the scope of our paper.

### 1.2. Related Works

There are two approaches for achieving authentication in wireless communication. One approach is to use “physical fingerprints”, which can be roughly divided into two categories: channel-based and radio frequency (RF) fingerprint-based schemes. The channel-based schemes [9,10,11,12,13] use CSI as a special kind of fingerprint that represents and discriminates different user identities. Another way of channel-based authentication is by generating an authentication vector according to both the CSI and the shared secret key. Paper [14] proposes an authentication scheme based on channel coding, where the shared key and CSI between two legitimate devices are combined against the adversary’s attack. A hybrid authentication protocol is proposed to integrate the CSI into the higher-layer security protocol without assuming a reliable reference channel estimation [15]. In [16,17], a key-based physical layer challenge-response authentication mechanism (PHY-CRAM) is studied, which doesn’t require any channel estimation or training. However, such schemes don’t provide integrity protection and cannot detect if the message has been manipulated or not. The RF fingerprint-based scheme [18,19,20] identifies a device according to the unique features of the waveform. RF fingerprint is caused by imperfections inherent in the hardware components. RF fingerprint-based authentication is more suitable for identity verification, while it is impractical to authenticate every symbol through this scheme. The other approach is called “authentication codes approach” [21,22,23,24]. This approach relies on modern cryptography and is a kind of computational security. Generally speaking, “authentication codes approach” belongs to coding theory and improves the chance of detecting deception by intentionally introducing redundant information in the transmitted message. “Authentication codes approach” reduces communication efficiency since extra authentication codes are transmitted. “Authentication codes approach”, such as the well-known message authentication codes (MAC) in cryptography, are the most popular solution to provide security services of data integrity and authentication in network communications. We take hash-based message authentication codes (HMAC) as an example to illustrate the principle of message authentication codes. The security of HMAC is based on the same shared root key and hash functions. Hash functions map from larger domains to smaller ranges and verify the integrity of the message while the shared root key verifies the identity of the transmitter. The security of MAC depends on the computational complexity and can be cracked if the malicious adversary with efficient computing resources. Especially in wireless communication, the transmitter and receiver share an unchanged root key due to the difficulty of key agreement and distribution. Wireless communication is more vulnerable to the malicious adversary.

### 1.3. Our Contributions

The focus of this paper is unconditional authentication in wireless communication, which is to study the performance of the authentication system if the malicious adversary with infinite computing resources. The security of the authentication methods discussed above are all based on computational complexity and is not an unconditional authentication from the perspective of information theory. It is difficult to achieve unconditional authentication like the well-known one-time pad for encryption. Fortunately, the unique and random characteristics of wireless channels can provide a source for generating true random keys, which is called physical layer key generation technology in most literature. Physical layer key generation has the potential to solve the key distribution problem, and thus makes unconditional authentication possible.

In this paper, we aim to propose an unconditional authentication framework based on one-time keys generated from the wireless channels. The framework can provide theoretical guidance for the authentication in wireless communication. Specifically, the contributions of this paper are summarized as follows:

- We derive the lower bound of unconditional authentication based on an unchanged key. We assume the adversary Mallory with unlimited computing resources and analyze the impersonation and substitution attacks. The probability of deception $\geq 2^{-\frac{1}{2}H\left(k\right)}$ is strictly derived from the perspective of information theory, where $H\left(k\right)$ is the entropy of the shared key. However, the lower bound $2^{-\frac{1}{2}H\left(k\right)}$ holds only for sending one authenticated message. The same key can not be used twice.
- Physical layer offered chain key (PHYLOCK) structure [25] is introduced to provide one-time keys for unconditional authentication so that we can achieve the lower bound $2^{-\frac{1}{2}H\left(k\right)}$. PHYLOCK can provide the root of trust for key generation and authentication. We conduct a security analysis of PHYLOCK and prove that PHYLOCK is more secure than the traditional physical layer key generation.
- Some conditions of unconditional authentication are listed. To realize the lower bound of unconditional authentication, encoding rules need to comply with some conditions. The conditions show that the length of the key and the authentication code are twice the length of the message.

The rest of this paper is organized as follows. Section 2 introduces the system and authentication model. Section 3 derives the lower bound of unconditional authentication based on an unchanged key. Section 4 points out that the pseudo-random key is not able to achieve unconditional authentication. Section 5 presents the structure and procedure of PHYLOCK and conducts security analysis of PHYLOCK. Following that Section 5 gives the definition and conditions of unconditional authentication. Section 6 concludes the paper and points out the significance, limitations, and future research of our paper.

## 2. System and Authentication Model

**Figure 1:**
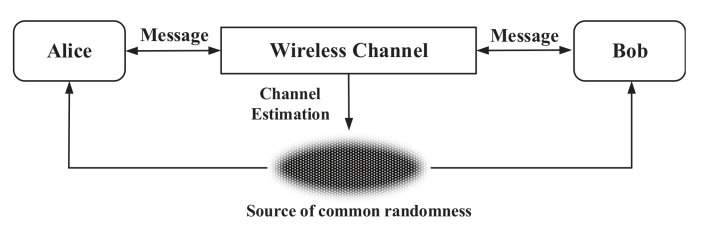
System model.

We consider a peer-to-peer wireless system depicted in Figure 1, where a legitimate transmitter (Alice) wants to send messages to the receiver (Bob). To overcome channel fading, accurate acquisition of CSI is essential to achieve spectrum and energy efficiency in wireless systems [26,27]. Alice and Bob obtain reciprocal CSI via various channel estimation methods. CSI is location-specific and time-varying due to path loss and channel fading [14,28]. It is difficult for an adversary to obtain information about the legitimate CSI as long as the distance between the malicious adversary and legitimate nodes is larger than half of the wavelength. CSI provides a source of common randomness that the malicious adversary has not or only partially, which can be used to generate secret keys shared only by Alice and Bob.

**Figure 2:**
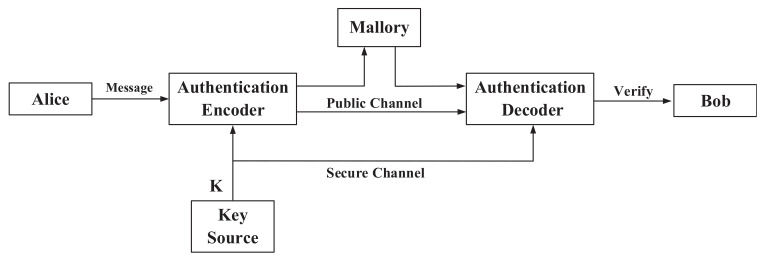
Authentication model.

Due to the broadcasting characteristics of wireless communication, wireless communication is vulnerable to various attacks. In this paper, we focus on the authentication problem that the legitimate receiver should be able to ensure the identity and the integrity of received messages, which is a major requirement of secure communications. In many scenarios, authentication is considered even more important than confidentiality since many messages might not be “secret”, but should be “authentic”. The authentication system is illustrated in Figure 2, where the message from Alice is authenticated by Bob and the reverse is the same. The authentication encoder outputs the authentication code *c*, which is a function of the secret key *k* and the message *m*. Then, the authentication code *c* together with the message *m* are sent to the receiver Bob. Bob uses the same encoder algorithm with the received message $\tilde{m}$ and the shared key *k* as input to generate the authentication code $\tilde{c}$. If $c=\tilde{c}$, Bob considers the received message as verified (i.e., the integrity test is successful). Otherwise, Bob judges that the message is not integral or from other illegal parties. We consider an active malicious adversary named Mallory that aims to deceive Bob to accept the fraudulent message. Attacks from Mallory can be divided into two types. One is the impersonation attack (successfully creating a fraudulent message) and the other is the substitution attack (successfully replacing a valid message with a fraudulent one). We consider a scenario in which Mallory has unlimited computing resources, which is different from the case of cryptography-based authentication, but indeed a major requirement for unconditional authentication. Furthermore, we assume Mallory knows everything about the system, except for the shared secret key. This is a well-known assumption in cryptography, known as Kerckhoff’s principle. It is equally reasonable to adopt Kerckhoff’s principle for authentication.

## 3. Lower Bound of Unconditional Authentication Based on an Unchanged Key

In this section, we analyze the lower bound of unconditional authentication based on an unchanged key, which is a basic analysis of Section 4 and Section 5.

To prevent impersonation and substitution attacks from Mallory, Alice encodes the message *m* using a key *k* to produce the authentication code *c*.

(1) $$c=f\left(m,k,v\right)$$

where *v* represents the initialization vector, which is usually a pseudo-random number. The initialization vector *v* guarantees that when Alice transmits the same message twice, the corresponding authentication codes are different. This makes it difficult for Mallory to perform a replay attack or obtain useful information about the authentication system. According to Kerckhoff’s principle [29], we assume Mallory knows everything about the authentication system, includes the encoding rules $f\left(\cdot ,\cdot \right)$, the message *m*, the initialization vector *v*, and the corresponding authentication code *c*, but does not know the shared key *k*. Since the initial vector *v* is known, in the following, we will simplify (1) as

(2) $$c=f\left(m,k\right)$$

Equation (2) simplifies the problem without losing generality. In some authentication schemes, the encrypted message $\bar{m}$ instead of the message *m* is used to generate the authentication code *c*. However, it does not affect the universality of Equation (2) because we assume Mallory already knows the message *m* and there is a one-to-one mapping between the encrypted messages $\bar{m}$ and the corresponding messages *m*. Then, Alice transmits the message and the corresponding authentication code together to Bob.

(3) $$y=\left(m;c\right)$$

Bob will use *c* to test the received message *m* for authenticity.

Mallory attempts to use a false message $m^{\prime }$ to launch impersonation or substitution attacks. Bob will calculate $c^{\prime }=f\left(m^{\prime },k\right)$, if $c\neq c^{\prime }$, Bob will discover Mallory’s deception. Mallory’s probability of escaping detection will be called $p_{0}$, which is the probability value when Mallory obtains the optimal strategy. In this paper, $p_{0}$ is the smallest probability of deception even though Mallory has unlimited computing resources, so we call $p_{0}$ the lower bound of unconditional authentication. Mallory can use the blind guessing scheme if Mallory does not have any prior information. For example, Mallory can successfully deceive Bob with probability $p_{0}\geq {\left|K\right|}^{-1}$ just by guessing a key at random with all $\left|K\right|$ keys equally likely. Another scheme is to guess the corresponding authentication code $c^{\prime }$ at random with all $\left|C\right|$ codes equally likely and $p_{0}\geq {\left|C\right|}^{-1}$. In fact, Mallory can always intercept the message between Alice and Bob and he can use the knowledge of *m* and *c* to restrict his guess, and thus improves the probability of deception.

**Figure 3:**
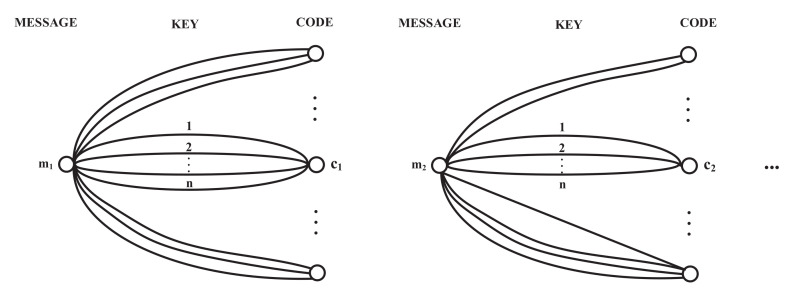
Diagram of message, key and code.

We first discuss the probability of deception informally when the key *k* keeps unchanged and Mallory obtains only one piece of the message *m* and the corresponding authentication code *c*. Mallory can use Equation (2) to learn the key *k*. Since Mallory has infinite computing power and Mallory can search the entire keyspace *K* to find the keys that satisfy Equation (2). In order to reduce the probability of being deceived, Bob must reasonably construct encoding rules $f\left(\cdot ,\cdot \right)$ so that Equation (2) has as many solutions as possible. The mapping among the message *m*, key *k*, and code *c* has a diagram like Figure 3, which depicts messages *m* as points in the left column and codes *c* as points in the right column. The lines directed from left to right are labeled by the key names 1,⋯,K to show how these keys encode each *m* into a code *c*. Suppose there are *n* solutions and the probability that Mallory will pick the correct key is $\frac{1}{n}$. As one might expect, Bob must use a large number of possible keys to provide many solutions to Equation (2). However, Mallory need not guess the correct key. Mallory still succeeds if

(4) $$f\left(m^{\prime },k_{0}\right)=f\left(m^{\prime },k\right)$$

where $m^{\prime }$ is the false message that Mallory wants to send to Bob, *k* is the correct key, $k_{0}$ is one of the *n* solutions satisfying $c^{\prime }=f\left(m^{\prime },k\right)$. Then, the probability of obtaining the correct $c^{\prime }$ is $\frac{n}{\left|K\right|}$. Therefore, the number of solutions *n* for every message can neither be too large nor too small. From the definition of $p_{0}$, we have

(5) $$p_{0}=\min\left\{\max\left\{\frac{1}{n},\frac{n}{\left|K\right|}\right\}\right\}$$

$\max\left\{\frac{1}{n},\frac{n}{\left|K\right|}\right\}$ means take the larger of the two probabilities. When $\frac{1}{n}=\frac{n}{\left|K\right|}$, $p_{0}$ takes the minimum value, that is $p_{0}={\left|K\right|}^{\frac{1}{2}}$.

Next, we will rigorously prove the lower bound of unconditional authentication from the perspective of information theory. Before the proof, we will list some natural restrictions on the behavior of Alice, Bob, and Mallory [30].

(a) Alice and Bob use the $\left|K\right|$ keys at random, equally likely and independent of the message *m*. Therefore, we have $\left|K\right|=2^{H\left(k\right)}$. Mallory is not subject to this restriction. He can use the keys in any way to help increase $p_{0}$.
(b) All $\left|C\right|$ coded messages are equally likely. In other words, every message is equally important. Alice and Bob do not have to protect some messages exclusively.
(c) Mallory picks $m^{\prime }$ at random from the $\left|C\right|-1$ coded messages different from $m^{\prime }$, all equally likely.
(d) Any different messages $m_{1}$, $m_{2}$ cannot be encoded into the same *c*, i.e., $f\left(m_{1},k_{1}\right)\neq f\left(m_{2},k_{2}\right)$, hold for all $k_{1}$, $k_{2}$, if $m_{1}\neq m_{2}$. This restriction only strengthens the lower bound of deception probability because there may be better strategies for Mallory if $f\left(m_{1},k_{1}\right)=f\left(m_{2},k_{2}\right)$.

Knowing how the message *m*, $m^{\prime }$ and key *k* are distributed, we can compute the joint probability $P\left(m,c,m^{\prime }\right)$. Define $p_{0}\left(m,c,m^{\prime }\right)$ to be the deception probability when Mallory substitutes a given $m^{\prime }$ for a given *m*, knowing *c*. The probability $p_{0}\left(m,c,m^{\prime }\right)$ depends on how Mallory uses *m*, *c*, $m^{\prime }$ to determine a false code $c^{\prime }$. Mallory knows the function $f\left(\cdot ,\cdot \right)$ and the key distribution. Then, Mallory can compute the conditional probability distribution $P\left(c^{\prime }|m,c,m^{\prime }\right)$ of the correct code $c^{\prime }=f\left(m^{\prime },k\right)$. Mallory maximizes his chance of success by selecting a $c^{\prime }$, which maximizes $P\left(c^{\prime }|m,c,m^{\prime }\right)$. Then, Mallory achieves $p_{0}\left(m,c,m^{\prime }\right)$ by

(6) $$p_{0}\left(m,c,m^{\prime }\right)=\underset{c^{\prime }}{Max}P\left(c^{\prime }|m,c,m^{\prime }\right))$$

Then, $p_{0}$ is the weighted average deception probability of all $p_{0}\left(m,c,m^{\prime }\right)$ with weight $P\left(m,c,m^{\prime }\right)$.

(7) $$p_{0}=\sum_{m,c,m^{\prime }}P\left(m,c,m^{\prime }\right)p_{0}\left(m,c,m^{\prime }\right)$$

Therefore, $p_{0}$ is optimal for Mallory when he adopts the best strategy. We now relate $p_{0}$ to the average uncertainty *U*, which Mallory has about the correct code $c^{\prime }$. By the definition of conditional entropy, we can calculate *U*

(8) $$\begin{array}{c} U=H\left(c^{\prime }|m,c,m^{\prime }\right)\\ =-\sum_{m,c,m^{\prime },c^{\prime }}P\left(m,c,m^{\prime },c^{\prime }\right)\log P\left(c^{\prime }|m,c,m^{\prime }\right) \end{array}$$

Next, we give **Lemma 1** to reveal the relationship between $p_{0}$ and *U*.

**Lemma** **1.** *if Mallory chooses optimal $c^{\prime }$ to make (6) holds, then*

(9) $$p_{0}\geq 2^{-U}$$

*The equality relationship in (9) holds if and only if all the possible $c^{\prime }$ for $m^{\prime }$ are equally likely and $P\left(m,c,m^{\prime }\right)\neq 0$. The equality in (9) means that there are exactly $2^{U}$ such $c^{\prime }$ for every given $\left(m,c,m^{\prime }\right)$.*

**Proof.**  Since ${\left(-\log x\right)}^{\prime \prime }=\frac{1}{x^{2}}>0$, for all x>0, the function −logx is concave. Then, we have Jenson’s inequality expressed as

(10) $$-\log\left(\sum_{i=1}^{n}\lambda_{i}x_{i}\right)\leq -\sum_{i=1}^{n}\lambda_{i}\log x_{i}$$

where $x_{i}>0,\sum_{i}\lambda_{i}=1,\;\lambda_{i}>0,\;i=1,2,\cdots ,n$. According to (6) and the concavity of the function −logx, we have

(11) $$\begin{array}{c} U=-\sum_{c^{\prime }}\sum_{m,c,m^{\prime }}P\left(m,c,m^{\prime },c^{\prime }\right)\log P\left(c^{\prime }|m,c,m^{\prime }\right)\\ \overset{\left(a\right)}{=}-\sum_{m,c,m^{\prime }}P\left(m,c,m^{\prime }\right)\log P\left(c^{\prime }|m,c,m^{\prime }\right)\\ \overset{\left(b\right)}{\geq }-\sum_{m,c,m^{\prime }}P\left(m,c,m^{\prime }\right)\log p_{0}\left(m,c,m^{\prime }\right)\\ \overset{\left(c\right)}{\geq }-\log\sum_{m,c,m^{\prime }}P\left(m,c,m^{\prime }\right)p_{0}\left(m,c,m^{\prime }\right) \end{array}$$

where step (a) is to sum on $c^{\prime }$, step (b) is because $p_{0}\left(m,c,m^{\prime }\right)\geq P\left(c^{\prime }|m,c,m^{\prime }\right)$, step (c) is based on the concavity of the function −logx and Jensen’s inequality. Now, we can get **Lemma 1** from (7) and (11).    □

The proof uses two inequalities in step (b) and step (c). Both must become equalities if equality holds in (9). The first inequality $p_{0}\left(m,c,m^{\prime }\right)\geq P\left(c^{\prime }|m,c,m^{\prime }\right)$ requires all possible $c^{\prime }$ to be equally likely for given *m*, *c*, $m^{\prime }$. In the discussion of Jensen’s inequality, equality requires all $\log p_{0}\left(m,c,m^{\prime }\right)$ terms to be equal to *U*.

We next bound $p_{0}$ in terms of the conditional entropy $H\left(k\right)$, which indicates the uncertainty of the key.

**Theorem** **1.** *Suppose (6) and restrictions (a), (b), (c), (d) all hold. Then,*

(12) $$p_{0}\geq 2^{-\frac{1}{2}H\left(k\right)}$$

**Proof.**  First note that $c^{\prime }$ is only determined by $c^{\prime }=f\left(m^{\prime },k\right)$ if $m^{\prime }$, *k* are given. Then, $c^{\prime }$ contains less information than $\left(m^{\prime },k\right)$.

(13) $$U=H\left(c^{\prime }|m,c,m^{\prime }\right)\leq H\left(m^{\prime },k|m,c,m^{\prime }\right)=H\left(k|m,c,m^{\prime }\right)$$

However, the conditional probability for *k* given *m*, *c*, $m^{\prime }$ depends only on *m*, *c*, so (13) becomes

(14) $$U\leq H\left(k|m,c\right)$$

Since the message and the key are independent and *c* is only determined by $c=f\left(m,k\right)$, we have

(15) $$H\left(k\right)=H\left(k|m\right)=H\left(k,c|m\right)$$

Due to the strong additivity of entropy, (15) becomes

(16) $$H\left(k\right)=H\left(k,c|m\right)=H\left(c|m\right)+H\left(k|m,c\right)$$

(14) and (16) provides

(17) $$U\leq H\left(k\right)-H\left(c|m\right)$$

and

(18) $$U=H\left(c^{\prime }|m,c,m^{\prime }\right)\leq H\left(c^{\prime }|m^{\prime }\right)$$

Due to restriction (c), $m^{\prime }$ is equally likely to be any one of the $\left|C\right|$ coded messages. Then, by restriction (b), *m* and $m^{\prime }$ have the same distribution, and finally

(19) $$U\leq H\left(c|m\right)$$

Now, compare (17) and (19). If $H\left(c|m\right)\geq \frac{1}{2}H\left(k\right)$, $U\leq \frac{1}{2}H\left(k\right)$ follows from (17). If $H\left(c|m\right)\leq \frac{1}{2}H\left(k\right)$, $U\leq \frac{1}{2}H\left(k\right)$ follows from (19). **Theorem 1** holds for both cases.    □

**Theorem 1** indicates Mallory can find $2^{\frac{1}{2}H\left(k\right)}$ solutions $k\in S\left(m_{i},c_{i}\right)=\left\{k|f\left(m_{i},k\right)=c_{i}\right\}$ and Mallory’s uncertainty about the key is $\frac{1}{2}H\left(k\right)$ with one message and code. When Mallory intercepts a second $m_{j},c_{j},j\neq i$, Mallory’s uncertainty about the key will drop rapidly because the real key fits both $k\in S\left(m_{i},c_{i}\right)$ and $k\in S\left(m_{j},c_{j}\right),i\neq j$. As Mallory intercepts more messages, the unchanged key will eventually be disclosed. Therefore, the key needs to be changed with each message sent.

## 4. Security Analysis under Pseudo-Random Key

Cryptography-based stream ciphers [31,32] are regarded as one of the technologies that generate constantly changing keys in wireless communication. However, it is impossible to achieve unconditional authentication using stream ciphers when Mallory has infinite computing power. In the following, we list two main reasons for this conclusion. The first reason is that more and more attack methods against stream ciphers appear, such as the algebraic attack [33], resynchronization attack [34], etc. The stream cipher is a computing security scheme and will become insecure when facing Mallory with infinite computing power. The second reason is that the stream cipher keys are generated by the initial key, and the entropy of the initial key is constant. From the perspective of information theory, as Mallory intercepts more and more messages and codes $\left(m_{i},c_{i}\right)$, the entropy of the initial key will gradually decrease and finally be cracked by Mallory. Therefore, only true random keys can achieve unconditional authentication.

## 5. Unconditional Authentication Based on PHYLOCK

In this section, we first introduce the structure and procedure of the physical layer offered chain key (PHYLOCK), which acts as a key generator and provides one-time keys for unconditional authentication. Then, we analyze the security of PHYLOCK and prove that PHYLOCK is a reliable secure key generator. Finally, we define unconditional authentication and list the conditions for achieving unconditional authentication under the framework of this paper.

### 5.1. The Structure and Procedure of PHYLOCK

**Figure 4:**
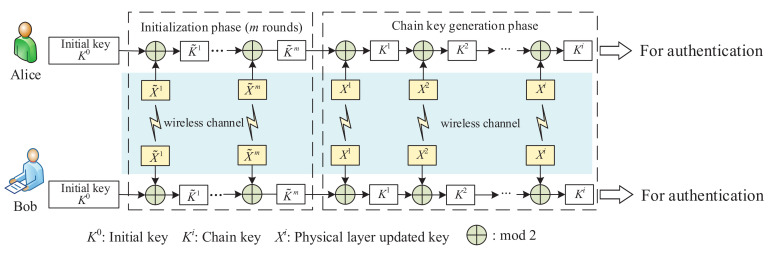
Structure of PHYLOCK.

**Figure 5:**
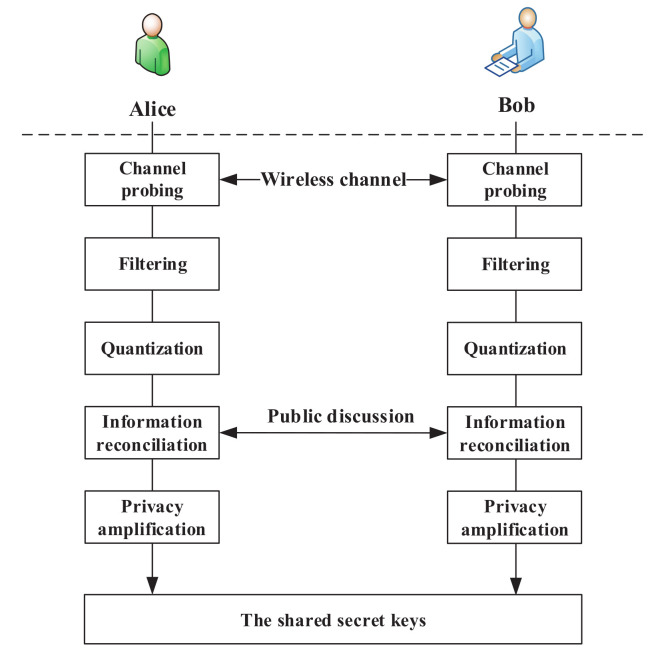
A block diagram of physical layer updated key generation.

The structure of PHYLOCK is shown in Figure 4, which includes three kinds of keys. The initial key $K^{0}$ is the start of PHYLOCK and provides the root of trust for Alice and Bob. $K^{0}$ is pre-stored by Alice and Bob through a secure channel and keeps unchanged in subsequent procedures. The physical layer updated key $X^{i}$ is generated from the wireless channel between Alice and Bob, which provides a source of common randomness, just as shown in Figure 5. It is almost impossible for Mallory who is located at a different place from Alice and Bob to obtain the same source of randomness for key generation. This is called the spatial decorrelation assumption in most key generation research exploiting channel randomness [35,36]. Figure 5 is the block diagram of the physical layer updated key generation. The procedure for extracting secret bits is generally divided into five phases. The first phase is channel probing. The purpose is to extract the same CSI between Alice and Bob, which is usually achieved by sending pilot symbols to each other. The second phase is filtering to obtain as consistent CSI as possible on the Alice and Bob sides. In the third phase, Alice and Bob input the CSI into the equal probability quantizer, respectively, and perform 1-bit quantization. In the information reconciliation phase, Alice and Bob should reconcile to a common key through public discussion while leaking as little information as possible. Finally, the privacy amplification phase applies universal hash functions to the reconciled information to ensure the shared secret key completely unknown to Mallory.

Chain key $K^{i},i>0$ is the one-time key used for unconditional authentication. Different from the traditional physical layer key generation [37,38], we perform XOR operation on the previous chain key $K^{i-1}$ and the physical layer newly generated key $X^{i},i>0$ to generate a new chain key $K^{i}$, which is expressed as

(20) $$K^{i}=K^{i-1}⊕X^{i}$$

The physical layer updated key $X^{i}$ continuously updates the chain key $K^{i-1}$. The chain key $K^{i}$ not only contains the information of the physical layer updated key $X^{i}$, but also the information of the last chain key. It is determined by all previous physical layer generated keys, thus making the chain highly secure. More security analysis of PHYLOCK is in Section 5.2.

PHYLOCK operates iteratively by physical layer updated keys continuously generated from the wireless channel. The initial key $K^{0}$ pre-set by Alice and Bob provides the root of trust for authentication. The initial key $K^{0}$ guarantees the trustworthiness of the physical layer updated key $X^{1}$ and chain key $K^{1}$, and thus the subsequent physical layer updated keys $X^{2},X^{3}\cdots$ and chain keys $K^{2},K^{3}\cdots$. The trust relationship between Alice and Bob is passed along with the iterative process. Before PHYLOCK officially generates chain keys, PHYLOCK first performs the initialization phase. The initialization phase iterates *m* rounds to mask the initial key $K^{0}$ and protect $K^{0}$ from being leaked. Then, the physical layer updated keys are generated along the data transmission. The key generation rate of PHYLOCK depends on the generation rate of physical layer updated keys. There are many physical layer key generation schemes in the previous work to increase the key generation rate [39,40]. Finally, the chain keys $K^{i},i=1,2,3\cdots$ generated by PHYLOCK are used for unconditional authentication. The procedure of PHYLOCK is summarized in Algorithm 1.
**Algorithm 1** The procedure of unconditional authentication based on PHYLOCK.**Input:** The initial key, $K^{0}$; The wireless channel state information, $CSI_{i}$; **Output:** The chain keys, $K^{i}$;
1:Alice and Bob perform initial authentication through the initial key $K^{0}$;2:Perform initialization phase of *m* rounds;3:Alice and Bob probe the wireless channel state information $CSI_{i}$ and generate physical layer updated key $X_{i}$, see Figure 5;4:Generate the chain keys: $K^{i}=K^{i-1}⊕X^{i}$;5:Use the chain keys for message authentication according to the encoding rules $f\left(\cdot ,\cdot \right)$;6:Repeat Steps 3–5;7:If the message authentication fails, Alice and Bob return to Step 2 and perform the initialization phase.


### 5.2. Security Analysis of PHYLOCK

The security of PHYLOCK is the basis for unconditional authentication. In this section, we conduct a security analysis of PHYLOCK. The security of PHYLOCK depends on the difficulty of Mallory to crack PHYLOCK. We consider three attack cases to illustrate the security of PHYLOCK. **Case 1:** Mallory obtains $X^{i}$ or $K^{i-1}$ to break PHYLOCK. This is possible, for example, if Mallory probes the same wireless channel and generates the same physical layer updated key $X^{i}$, or guessed the correct chain key $K^{i-1}$ according to the current message. **Case 2:** Mallory gets both $X^{i}$ and $K^{i-1}$ to break PHYLOCK. **Case 3:** Mallory knows all $X^{i}$ and $K^{i}$, and attempts to recover the initial key $K^{0}$. **Case 3** is similar to the known-plaintext attack in stream cipher and can be regarded as the worst attack case for PHYLOCK. The difficulty of these attacks is ascending. In addition to these three attack cases, we also discuss and simulate the performance of PHYLOCK under correlated channel attacks.

#### 5.2.1. Analysis for Case 1

The security under **Case 1** means that Mallory can not reduce the uncertainty about $K^{i}$ by knowing $K^{i-1}$ or $X^{i}$. We need to prove the following equation holds:

(21) $$H\left(K^{i}\right)=H\left(K^{i}|K^{i-1}\right)$$

(22) $$H\left(K^{i}\right)=H\left(K^{i}|X^{i}\right)$$

**Proposition** **1.** *For the chain key $K^{i}=\left(k_{1}^{i}k_{2}^{i}\cdots k_{r}^{i}\right)$ and physical layer updated key $X^{i}=\left(x_{1}^{i}x_{2}^{i}\cdots x_{r}^{i}\right)$, where r is the key length, $x_{j}^{i}\in \left\{0,1\right\},j=1,2,\cdots ,r$ is an independent and identically distributed (i.i.d) random variable whose probability distribution satisfies $\mathrm{Pr}\left(x_{j}^{i}=1\right)=\mathrm{Pr}\left(x_{j}^{i}=0\right)=0.5$, then we get $H\left(K^{i}\right)=H\left(K^{i}|K^{i-1}\right)=r$ bits, $H\left(K^{i}\right)=H\left(K^{i}|X^{i}\right)=r$ bits.*

**Proof.**  First, we calculate the probability distribution of $\mathrm{Pr}\left(K^{i}\right)$.

(23) $$\begin{array}{c} \mathrm{Pr}\left(k_{j}^{i}=1\right)=\mathrm{Pr}\left(k_{j}^{i-1}⊕x_{j}^{i}=1\right)\\ =\mathrm{Pr}\left(k_{j}^{i-1}=1\right)\mathrm{Pr}\left(x_{j}^{i}=0\right)+\mathrm{Pr}\left(k_{j}^{i-1}=0\right)\mathrm{Pr}\left(x_{j}^{i}=1\right)\\ =0.5\left(\mathrm{Pr}\left(k_{j}^{i-1}=1\right)+\mathrm{Pr}\left(k_{j}^{i-1}=0\right)\right)=0.5 \end{array}$$

Similarly, we can get $\mathrm{Pr}\left(k_{j}^{i}=0\right)=0.5$. Next, we calculate the conditional probability distribution $\mathrm{Pr}\left(K^{i}|K^{i-1}\right)$.

(24) $$\begin{array}{c} \mathrm{Pr}\left(k_{j}^{i}=1|k_{j}^{i-1}\right)\\ =\mathrm{Pr}\left(k_{j}^{i-1}⊕x_{j}^{i}=1|k_{j}^{i-1}=0\right)\mathrm{Pr}\left(k_{j}^{i-1}=0\right)+\\ \mathrm{Pr}\left(k_{j}^{i-1}⊕x_{j}^{i}=1|k_{j}^{i-1}=1\right)\mathrm{Pr}\left(k_{j}^{i-1}=1\right)\\ =0.5\left(\mathrm{Pr}\left(x_{j}^{i}=1\right)+\mathrm{Pr}\left(x_{j}^{i}=0\right)\right)=0.5 \end{array}$$

Similarly, we can get $\mathrm{Pr}\left(k_{j}^{i}=0|k_{j}^{i-1}\right)=0.5$. Since $\mathrm{Pr}\left(K^{i}\right)=\mathrm{Pr}\left(K^{i}|K^{i-1}\right)$, $K^{i}$ and $K^{i-1}$ are independent of each other and (21) holds. Following the same derivation process, we can get $H\left(K^{i}\right)=H\left(K^{i}|X^{i}\right)=r$ bits. **Proposition 1** shows that Mallory can not decrease $H\left(K^{i}\right)$ by knowing $K^{i-1}$ or $X^{i}$. This means that even if Mallory somehow obtains $K^{i-1}$ or $X^{i}$, he still can not obtain any information about $K^{i}$.  □

#### 5.2.2. Analysis for Case 2

From the security analysis for **Case 1**, it is easy to find that Mallory must obtain both $K^{i-1}$ and $X^{i}$ to break $K^{i}$. However, due to the chain structure of PHYLOCK, the leakage of $K^{i}$ does not affect other chain keys.

**Proposition** **2.** *If Mallory gets both $X^{i}$ and $K^{i-1}$, Mallory can only obtain $K^{i}$, but cannot get anything about the previous chain keys $K^{i-2},K^{i-3}\cdots ,i>2$ and the following chain keys $K^{i+1},K^{i+2}\cdots ,i>2$.*

**Proof.**  (25) $$\begin{array}{c} H\left(K^{i+1}|X^{i}K^{i-1}K^{i}\right)\\ =H\left(K^{i}⊕X^{i+1}|X^{i}K^{i-1}K^{i}\right)\\ =H\left(K^{0}⊕X^{1}⊕\cdots ⊕X^{i+1}|K^{0}⊕X^{1}⊕\cdots ⊕X^{i}\right)\\ =H\left(X^{i+1}\right)=r\;bits \end{array}$$

Similarly,

(26) $$\begin{array}{c} H\left(K^{i-2}|X^{i}K^{i-1}K^{i}\right)\\ =H\left(K^{i-1}⊕X^{i-2}|X^{i}K^{i-1}K^{i}\right)\\ =H\left(K^{i-1}⊕X^{i-2}|K^{i-1}\right)\\ =H\left(K^{0}⊕X^{1}⊕\cdots ⊕X^{i-2}|K^{0}⊕X^{1}⊕\cdots ⊕X^{i-1}\right)\\ =H\left(X^{i-2}\right)=r\;bits \end{array}$$

It can be derived from (25) and (26) that the disclosure of a certain chain key $K^{i}$ does not affect the security of previous and following chain keys. Every chain key is updated by the corresponding physical layer updated key. Therefore, Mallory needs to know all the channel information state (CSI) to break PHYLOCK. However, it is difficult for Mallory to obtain the same CSI as Alice and Bob.   □

#### 5.2.3. Analysis for Case 3

In attack **Case 3**, we assume Mallory obtains all the $K^{1},K^{2},\cdots ,K^{i}$. This is the worst attack case for PHYLOCK and Mallory attempts to recover $K^{0}$. If $K^{0}$ can be recovered, then PHYLOCK can be regarded as completely cracked by Mallory.

**Proposition** **3.** *If Mallory gets all $X^{1},X^{2},\cdots ,X^{i}$ and $K^{1},K^{2},\cdots ,K^{i}$, he still can not get any information about $K^{0}$ because of the initialization phase of PHYLOCK.*

**Proof.**  The entropy of $K^{0}$ under **Case 3** is calculated as follows

(27) $$\begin{array}{c} H\left(K^{0}|K^{1}\cdots K^{i}X^{1}\cdots X^{i}\right)\\ =H\left(K^{1}⊕X^{1}⊕{\tilde{X}}^{m}⊕{\tilde{X}}^{m-1}⊕\cdots ⊕{\tilde{X}}^{1}|K^{1}\cdots K^{i}X^{1}\cdots X^{i}\right)\\ =H\left({\tilde{X}}^{m}⊕{\tilde{X}}^{m-1}⊕\cdots ⊕{\tilde{X}}^{1}\right)=r\;\mathrm{bits}=H\left(K^{0}\right) \end{array}$$

Mallory can not decrease the entropy of $K^{0}$ because the initialization phase masks $K^{0}$ well.   □

#### 5.2.4. Analysis for Correlated Channel Attack

Spatial decorrelation is essential to the security of physical layer key generation. However, some studies have shown that wireless channels may lose the characteristics of spatial decorrelation when Eve or Mallory is close enough to legitimate nodes or launches a pilot attack [41,42]. Traditional physical layer key generation schemes are vulnerable to correlated channel attacks. Fortunately, the proposed PHYLOCK can resist correlated channel attacks well. We assume Eve or Mallory already know a certain $K^{i}$ and can generate keys $X_{E}^{i+j},j=1,2,\cdots ,n$ from wireless channels that are highly correlated to the physical layer update keys $X^{i+j},j=1,2,\cdots ,n$ in the subsequent. The key bit error probability (BER) [43] $P_{AE}$ between $X_{E}^{i+j},j=1,2,\cdots ,n$ and $X^{i+j},j=1,2,\cdots ,n$ can be given according to the correlation coefficient. To simplify the analysis, we directly set the value of $P_{AE}$ and keep unchanged with the iteration of PHYLOCK. After *n* iterations of PHYLOCK, the BER between $K^{i+n}$ and $K_{E}^{i+n}$ can be expressed as

(28) $$BER\left(K^{i+n},K_{E}^{i+n}\right)=\sum_{k=1,3,\cdots ,2\left\lceil \frac{n}{2}\right\rceil -1}C_{n}^{k}P_{AE}^{k}\cdot {\left(1-P_{AE}\right)}^{n-k}$$

$k=1,3,\cdots ,2\left\lceil \frac{n}{2}\right\rceil -1$ is because the keys at the corresponding locations between $K^{i+n}$ and $K_{E}^{i+n}$ are inconsistent only when an odd number of key errors occur.

**Figure 6:**
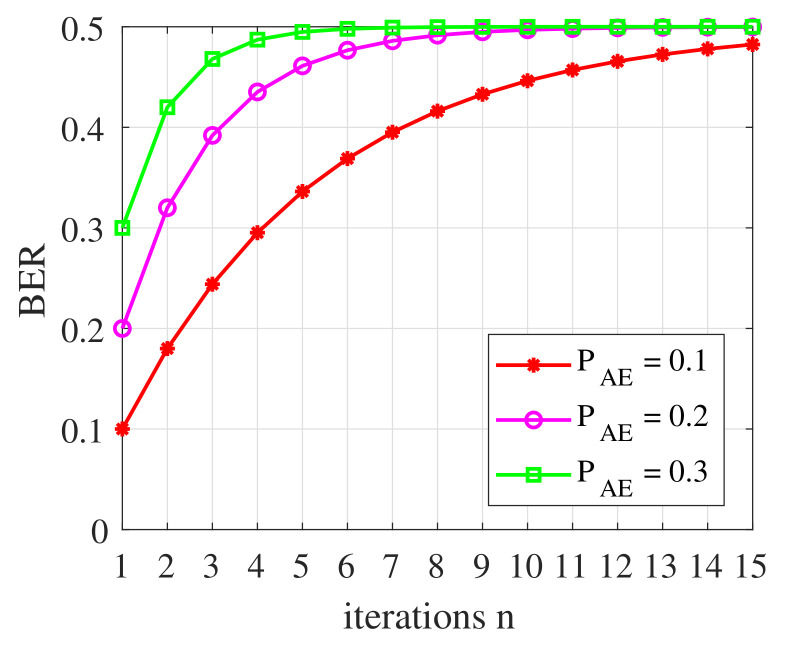
BER between $K^{i+n}$ and $K_{E}^{i+n}$ versus the iterations of PHYLOCK.

Figure 6 shows the BER between $K^{i+n}$ and $K_{E}^{i+n}$ versus the iterations of PHYLOCK. As the number of iterations *n* increases, the BER increases to 0.5 rapidly even if $P_{AE}$ is small due to the strong correlation between the legitimate channel and illegitimate channel. This is because the iterative structure of PHYLOCK makes inconsistent keys constantly accumulate. BER approaches 0.5 after 10 iterations, which means that Eve or Mallory can obtain nothing about $K^{i+n}$ even if the correlated channel attack is still ongoing. For traditional physical layer key generation schemes, they will always be threatened by correlated channel attacks. From the perspective of information theory, the iterative structure of PHYLOCK makes Eve or Mallory’s uncertainty about the legitimate channel continue to accumulate.

### 5.3. Definition and Conditions of Unconditional Authentication

The analysis of the above two sections shows that we can generate one-time keys by PHYLOCK that makes unconditional authentication possible. In this section, we give the definition and framework of unconditional authentication in wireless communication and list the conditions for achieving unconditional authentication.

**Definition** **1.** *For a wireless authentication system with appropriate encoding rules $f\left(\cdot ,\cdot \right)$ and one-time keys generator PHYLOCK, if Mallory has unlimited computing resources and his probability of deception $p_{0}$ satisfies $p_{0}=2^{-\frac{1}{2}H\left(k\right)}$, then the system achieves unconditional authentication.*

Next, we list the conditions that need to be met to achieve unconditional authentication. Note that realization of unconditional authentication is when equality holds in (12). If equality is to hold in (12), all the inequalities used in proving **Theorem 1** must become equalities. We now review these inequalities to obtain requirements.

We first give three conditions that are most easily stated in the diagram, Figure 3.

(i) Every pair of bundles, from $m_{1}$ to $c_{1}$ and $m_{2}$ to $c_{2}$, with $m_{1}\neq m_{2}$, has only one key in common.
(ii) Every bundle contains $2^{\frac{1}{2}H\left(k\right)}$ keys.
(iii) There are $2^{\frac{1}{2}H\left(k\right)}$ bundles at each *m*.

To prove (i), (ii), (iii), begin with (13). The equality $H\left(c^{\prime }|m,c,m^{\prime }\right)=H\left(m^{\prime },k|m,c,m^{\prime }\right)$ means that there is only one key *k* satisfied $c^{\prime }=f\left(m^{\prime },k\right)$ under the condition $\left(m,c,m^{\prime }\right)$. If for some $\left(m,c,m^{\prime }\right)$, more than one key *k* satisfied $c^{\prime }=f\left(m^{\prime },k\right)$, then the conditional entropy about $c^{\prime }$ is higher. Therefore, we have condition (i) hold.

The equation $p_{0}=2^{-\frac{1}{2}H\left(k\right)}$ means that there are $2^{\frac{1}{2}H\left(k\right)}$ possibles keys to satisfy $c=f\left(m,k\right)$, which proves (ii) every bundle contains $2^{\frac{1}{2}H\left(k\right)}$ keys. We can also derive condition (ii) from another perspective. The equality in (9) requires that the keys in any bundle from *m* to *c* be distributed equally over $2^{U}=2^{\frac{1}{2}H\left(k\right)}$ images $c^{\prime }$ of any $m^{\prime }$. Each of these keys leads from $m^{\prime }$ to a different $c^{\prime }$(by (i)). Then, the bundle *m* to *c* has $2^{\frac{1}{2}H\left(k\right)}$ keys. Now, (iii) follows from (ii) because there are only $2^{H\left(k\right)}$ keys. Conditions (ii) and (iii) also guarantee $H\left(c|m\right)=\frac{1}{2}H\left(k\right)$, which is needed for equality in (17) and (19).

(i) requires a pair of keys $\left(k_{1},k_{2}\right),k_{1}\neq k_{2}$ to belong to at most one bundle. The number of pairs having a common bundle in one cluster is $C_{2^{\frac{1}{2}H\left(k\right)}}^{2}*2^{\frac{1}{2}H\left(k\right)}$. The number of clusters is equal to the number of messages *M*. So the number of pairs of keys having a common bundle is $C_{2^{\frac{1}{2}H\left(k\right)}}^{2}*2^{\frac{1}{2}H\left(k\right)}*M$. This number must be no larger than the unrestricted number of pairs of keys $C_{2^{H\left(k\right)}}^{2}$, thus

(29) $$\begin{array}{c} C_{2^{\frac{1}{2}H\left(k\right)}}^{2}*2^{\frac{1}{2}H\left(k\right)}*M\leq C_{2^{H\left(k\right)}}^{2}\\ M\leq 2^{\frac{1}{2}H\left(k\right)}+1 \end{array}$$

Follows (iii), the length of the code is expressed as $C=M*2^{\frac{1}{2}H\left(k\right)}$.

(iv) The length of the message *m* is no more than half the length of the key and the length of the code.

From (iv) we know that $p_{0}=2^{-\frac{1}{2}H\left(k\right)}$ can only be achieved by severely restricting the length of messages *m*.

Last but most importantly, the key has to be changed for every piece of the message because only one key fits both $c=f\left(m,k\right)$ and $c^{\prime }=f\left(m^{\prime },k^{\prime }\right)$ [by (i)] and a second message with the same key would disclose the key.

(v) The key needs to be changed through our proposed PHYLOCK key generation architecture for every piece of the message.

In our authentication model, Mallory can always intercept the current message and code. Then, Mallory can search the whole key space according to $c=f\left(m,k\right)$ and thus reduce the entropy of the key. According to **Definition 1**, the key entropy is halved by Mallory based on a pair of $\left(m,c\right)$. If the key keeps unchanged, then Mallory would disclose the key by a second $\left(m,c\right)$. Therefore, we must make (v) hold to achieve unconditional authentication. The strict conditions we listed above imply that unconditional authentication is impractical, but we can compromise among three conflicting goals: small $p_{0}$, small $\left|K\right|$, and large $\left|M\right|$.

**Figure 7:**
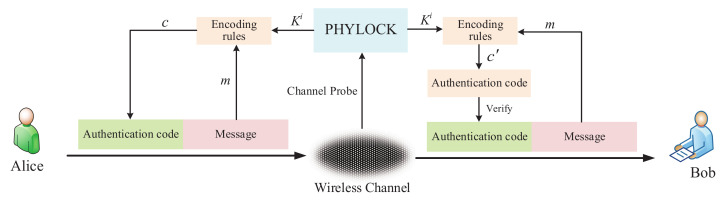
The framework of wireless unconditional authentication.

Finally, we give the framework of wireless unconditional authentication in Figure 7. Alice and Bob generate one-time keys through PHYLOCK, the structure of which is depicted in Figure 4. The chain keys $K^{i}$ generated by PHYLOCK together with the message *m* generate authentication code *c* according to the encoding rules. The encoding rules must meet the conditions we discussed above. The data transmission and the procedure of authentication are integrated together.

## 6. Conclusions

This paper analyzes unconditional authentication based on the physical layer offered chain key (PHYLOCK) in wireless communication. The chain key is generated through a chain structure iteratively. The initial key provides the root of trust and the chain key is updated by the physical layer updated key generated from the wireless channel. We conduct a security analysis of PHYLOCK and proves that it can provide one-time keys for unconditional authentication. Then, we analyze unconditional authentication from the perspective of information theory and the encoding rules that should be followed for unconditional authentication. However, the unconditional authentication framework we proposed is only applicable to wireless communications and the chain key rate depends on the entropy of the wireless channel and further limits the message rate. It can be inferred from the strict requirements of unconditional authentication that our framework is impractical, but provides theoretical guidance. The issue of authentication in the presence of wireless channel noise and channel coding is not addressed in this paper, which is our future research.
